# Quality and safety of in-hospital care for acute medical patients at weekends: a qualitative study

**DOI:** 10.1186/s12913-018-3833-z

**Published:** 2018-12-29

**Authors:** Elizabeth Sutton, Julian Bion, Cassie Aldridge, Amunpreet Boyal, Janet Willars, Carolyn Tarrant

**Affiliations:** 1 Department of Health Sciences, University of Leicester, Leicester, UK; 2 University Hospitals Birmingham, Birmingham, UK

**Keywords:** Hospital, Weekend, Quality of care, Qualitative

## Abstract

**Background:**

The increased mortality risk associated with weekend admission to hospital (the ‘weekend effect’) has been reported across many health systems. More recently research has focused on causal mechanisms. Variations in the organisation and delivery of in-hospital care between weekends and weekdays have been identified, but this is not always to the detriment of weekend admissions, and the impact on mortality is uncertain. The insights of frontline staff and patients have been neglected. This article reports a qualitative study of patients and clinicians, to explore their views on quality and safety of care at weekends.

**Methods:**

We conducted focus groups and interviews with clinicians and patients with experience of acute medical care, recruited from three UK hospital Trusts. We analysed the data using a thematic analysis approach, aided by the use of NVivo, to explore quality and safety of care at weekends.

**Results:**

We held four focus groups and completed six in-depth interviews, with 19 clinicians and 12 patients. Four threats to quality and safety were identified as being more prominent at weekends, relating to i) the rescue and stabilisation of sick patients; ii) monitoring and responding to deterioration; iii) timely accurate management of the therapeutic pathway; iv) errors of omission and commission.

**Conclusions:**

At weekends patients and staff are well aware of suboptimal staffing numbers, skill mix and access to resources at weekends, and identify that emergency admissions are prioritised over those already hospitalised. The consequences in terms of quality and safety and patient experience of care are undesirable. Our findings suggest the value of focusing on care processes and systems resilience over the weekends, and how these can be better supported, even in the limited resource environment that exists in many hospitals at weekends.

**Electronic supplementary material:**

The online version of this article (10.1186/s12913-018-3833-z) contains supplementary material, which is available to authorized users.

## Background

Patients admitted to hospital at weekends have been shown in many studies to be at higher risk of death and poorer outcomes than those admitted on weekdays, the so called ‘weekend effect’ [1, 2]. This has stimulated the UK government to prioritise the development of ‘seven day services’, a flagship health policy intended to improve care provision, safety, quality and outcomes. A central component of this policy rests on increasing the presence of clinical staff in the hospital at weekends, particularly senior doctors (consultants) [3] and junior doctors [4]. The underlying presumption is that increased mortality at weekends is related in some way to suboptimal input by medical staff, and that increasing the number of doctors and changing their working patterns will drive improvements in mortality outcomes for patients admitted at weekends [5].

Despite strong evidence that patients admitted at weekends are at higher risk of death than those admitted on weekdays, studies have failed to find associations between structural features of weekend service delivery including staffing levels, and mortality rates [2]. Similarly, implementation of seven day service standards has not yet been found to be associated with improved clinical outcomes [6, 7]. In addition, the focus on weekends in a resource-constrained system could have a negative impact on services provided during the week [6], casting doubt on the value of large scale service reorganisation for reducing mortality rates. Indeed, recent research has shown that fewer patients are admitted at weekends [7], while those who are admitted are sicker [7–11]. Only a small proportion of deaths in patients admitted at weekends may, in fact, be avoidable [12], and mortality alone is an insufficient indicator of care quality. Susceptibility to detriments in quality of care also varies between patient groups at different points in the care pathway [13]. However, the focus on mortality has to some extent obscured questions about how features of weekend care impact on patient and staff experience of care quality. While some studies have shown that the availability or performance of certain therapeutic procedures are delayed at weekends (for example, ‘door to balloon’ times for percutaneous coronary interventions for myocardial infarction) [14, 15], and can be improved by focusing on care processes [16, 17], others find variation in care processes throughout the week [18–20], some report better care at weekends [21] and one study reports better patient experience of emergency department care at weekends [22]. Despite this we found no *qualitative* evidence on the features of weekend care delivery and how these impacted on quality and safety from the perspective of staff and patients. Given this absence of evidence, it would seem sensible to seek the views of those most directly involved: the patients and front-line clinical staff who have experienced hospital care at weekends and on weekdays. Our study focused on the acute medical (non-operative) pathway, exploring staff and patients’ perceptions of quality and safety for acute medical patients admitted to or experiencing a stay in hospital at the weekend.

## Methods

This study was part of a wider study into the impact of specialist intensity on quality and outcomes of care at weekends (https://www.hislac.org/), which included a systematic literature review [23], quantitative studies of the association between specialist intensity and mortality [2], and case studies of quality of care across 20 hospitals assessed via observational research [24] and case note review [25]. To inform the wider study, we conducted a sub-study involving focus groups and interviews with clinicians and patients from three UK hospital trusts (in the Midlands, North East, and South West). This sub-study aimed to develop understanding of the mechanisms related to quality and safety of care which could contribute to poorer patient outcomes for acute medical patients at weekends.

Participating clinicians were recruited through the local project lead for the main study at each trust and were purposively sampled to include medical and nursing staff with a mix of roles and experience. In one location, it was difficult to find a suitable time for staff to participate in a focus group and they opted to take part in interviews instead. Patients were recruited through two existing acute care Patient and Public Involvement (PPI) groups, and through face-to-face recruitment of patients and relatives during their stay on the acute medical wards; this enabled us to involve both ‘expert’ patient representatives who had had multiple admissions and extensive experience of hospital care, and patients who had recent experience of a hospital stay over the weekend. PPI members were involved in focus groups but interviews were used with the latter group of patients to enable them to reflect on individual experiences. The group discussions were facilitated by JW, with ES and CA or AB taking notes and clarifying questions. Each discussion lasted between 1.5–2 h. Interviews were conducted by JW and CA and lasted for a maximum of an hour. The participants had no previous relationship with the researchers. A pre-piloted topic guide (Additional files 1, 2, 3, 4) was used for both interviews and focus groups to stimulate discussion and debate. During interviews and focus groups we asked people to discuss features of care delivery, based on evidence from the literature and from their own experiences, and how these impacted on quality and safety of care at weekends. All interviews and focus groups took place in in specifically allocated rooms within the hospitals. The following features of services delivery were discussed in focus groups in relation to weekend quality of care: staffing levels and skill mix; day-to-day continuity of individual staff and care teams; availability of diagnostic, therapeutic and allied services; and availability of community services.

All discussions were audio-recorded, transcribed verbatim, and anonymised. A thematic analysis approach [26] was used to analyse data from interviews and focus groups, aided by NVivo 11 [27]. Data were initially open-coded by ES using a descriptive coding frame (Additional file 5), and higher order themes were developed through close inspection of codes and discussions between members of the research team until no new themes emerged. We also used charts and narrative summaries to develop our interpretation.

## Results

We conducted four focus groups, two with clinicians (*n* = 15), and two with patients (*n* = 10), along with two patient interviews and four clinician interviews (Table 1).

**Table 1:** Focus group and interview participants

	Hospital 1	Hospital 2	Hospital 3	Total
Consultants	3	3	1	7
Nursing staff	4	2	1	7
Registrar/junior doctor	1	2	2	5
Patients	7	3	2	12

We aimed to identify patient and staff experiences of quality and safety at weekends. We describe the findings under four themes: rescue and stabilisation of sick patients, monitoring and responding to deterioration, timely and effective management of the therapeutic pathway; and resilience and risk of error.

### Rescue & stabilisation of sick patients

The rescue and stabilisation of sick patients is a core imperative of acute care [28, 29]. The routines of acute medicine were seen by participants as being organised first and foremost around responding to acutely sick patients. Focus group participants argued that this meant that, in the context of reduced staffing resources at the weekend, those patients who were most acutely unwell were prioritised for attention. These were particularly those patients coming in through Accident & Emergency (A&E) with life-threatening conditions. Clinicians described systems in place in their hospitals to enable review and categorisation of patients via emergency department triage and medical review of all newly admitted patients on acute medical units (AMUs), as well as systems to flag acutely unwell and unstable patients who would be in hospital over the weekend. The categorisation of patients as ‘sick’ provided a means of ordering and prioritising, enabling the allocation of the scarce resources at the weekend to those who were seen as having the most urgent and legitimate need for medical intervention. Even in cases where staffing levels were much lower at weekends, where senior doctors were few in number, and diagnostic and allied services were limited, participants felt that very sick patients would still get prompt and effective care. This was reflected in some patients’ accounts of their experiences of care at weekends.

*I think sick patients, from what I’ve seen actually, there’s no difference from the level of care they get [at the weekend], they’re still picked up as being the sickest, still seen first (Junior doctor interview)*

*I think, from an emergency care point of view […] at a weekend, the care was excellent, because I think it is [excellent] when it’s a dire emergency. I think my admission during the week was actually less efficient and worse than it was, the one at the weekend. (Patient focus group)*

Participants did recognise, however, that lack of staff with specific expertise at the weekend impacted on being able to provide quick, condition specific, treatment to rescue acutely unwell patients.

*There are some fairly urgent procedures […] that don’t get done as quickly at a weekend. […] We try and get central lines put in but because of the restrictions as to where these can be put in and how they’re put in and who can do them now, people won’t get their lines and their antibiotics and their treatment done as quickly as they would otherwise have done. (Clinician focus group)*

Clinicians also felt that a lack of continuity of staff on wards, including more locum and agency staff working at weekends, meant that simple tasks could be frustrated by a lack of tacit knowledge about how things worked and where equipment could be found, slowing down doctors’ efforts to manage sick patients.

*Going back to the base [medical] wards, sometimes you’ll turn up to see a sick patient and […] you can go to a ward and ask where something like a blood bottle is or a blood gas syringe and nobody on the whole ward will know, and it can make your job technically [difficult] – it can double the amount of time everything takes. (Clinician focus group)*

Despite some challenges at the weekend in responding quickly to serious and urgent medical problems, the depleted resources within the hospital at weekends were seen to impact less on those categorised as the ‘sickest’. Prioritisation decisions, which favoured the acutely sick, were, however, seen as resulting in a lower capacity to attend to patients admitted through A&E, or within the hospital, who were less acutely unwell.

*I think it’s the less sick probably have a worse experience [at weekends] because they’re being pushed down the list, and waiting longer. […] If they’re not in that top tier of urgency, then they won’t get things. (Junior doctor interview)*

### Monitoring and responding to deteriorating patients

Patients who were not deemed to be acutely sick, but who deteriorated over the weekend, were seen as particularly vulnerable to delays in detection and response. This risk arose when reduced staffing levels and skill mix across staff in all types of roles in the hospital resulted in a decrement in monitoring and a reduced likelihood that deterioration would be picked up opportunistically.

*You’ve got less experienced people, you’ve got more bank staff, you’ve got less doctors, you’ve got less – it’s the whole thing. (Patient focus group)*

*It’s about spotting the issues where people are becoming more poorly and if there’s a lower staff level and perhaps senior staff level, perhaps some things just don’t get picked up as quickly. (Patient focus group)*

*The frequency with which patients are seen by anybody working in the hospital. So, for instance, as you were saying, that often pharmacists can pick up on things that – the physios maybe brought out things to you when they’ve been to see them, and the nursing staff compile things and offer up novel observations. But, during the weekend, people are just seen much more infrequently [and…] you don’t have the frequency of other people seeing them, just to pick up on the little things (Clinician focus group)*

Participants highlighted a general shift from scheduled senior reviews of all patients during the week, and higher levels of presence on the ward of all grades of doctor, to an approach at weekends that was oriented more towards selective review and responding reactively when problems arose. Participants identified that opportunities for senior doctors to have informal conversations about patients with nurses and junior doctors, and to ‘keep an eye on patients’ were less likely to arise at weekends due to the lower levels of staffing, and systems for weekend working. This meant fewer opportunities to identify and pre-empt problems.

*R1: During a week […] nursing staff can come up to you and say, ‘So-and-so’s not looking that good. […] At the weekend, because you might be on a different ward, it all goes through kind of a bleep system and then …*

*R2: So the casual sort of conversation that you might have as you’re passing […] in a corridor, you wouldn’t get the opportunity to have (Clinician focus group).*

The allocation of scarce senior medical staff time to managing prioritised sick patients at the weekend meant that junior doctors were seen as holding more responsibility for responding to deteriorating patients, often with limited supervision. In the experience of junior doctors, this meant that escalation of deteriorating patients for more senior review could incur significant delay, which could be compounded by difficulties for junior doctors in making a case for the legitimacy of prioritising these patients.

*A specific example that I’ve had at a weekend where it was really quick to get a consultant review in the morning, the plan was in place, everything was fine, and that patient really deteriorated throughout the day and I really struggled to get a senior review on this patient. I was literally calling people and they were saying ‘I’m too busy with patients in resus [resuscitation area]’ or whatever (Clinician group 2).*

Discontinuity of clinicians over the weekend was also seen as detrimental to the ability to respond appropriately to deteriorating patients.

*The advantage of the weekdays is it’s a team in that ward that knows those patients, so if anything goes wrong, they know the patient, whereas on a weekend, you are going to call someone from somewhere who doesn’t know these patients, who’s going to need to come in and fix whatever it is that is wrong. (Clinician focus group)*

There was a sense that deteriorating patients were less likely to be proactively identified and managed at the weekend, meaning opportunities for intervention were missed.

*So when you come in as the team on the Monday, ‘oh, this patient became very unwell, oh, they’re still quite unwell, oh, we perhaps missed a window of opportunity’ (Junior doctor interview).*

### Therapeutic pathway flow – Diagnosing, moving treatment on, resolving and discharging

A further imperative of acute medical care, and a core function of AMUs, is to label and ‘dispose’ [30] of patients i.e. to undertake decision-making about the likely clinical problem, to initiate suitable treatment or care, and to direct patients onwards to an appropriate destination, whether this is a ward placement, or discharge. Despite recognition of the importance of maintaining flow, for example by using discharge teams, there was a strong view that stretched resources at the weekend meant that the pace of care dramatically slowed at weekends.

One factor in this slowing down of flow along pathways was the availability of senior medical input, and particularly specialist input, in decision-making. Not only was there a tension for the weekend consultants in balancing the work of moving less acute patients onwards through the system against the pressing need to rescue and respond to acutely sick patients, but at weekends the specialty expertise required for good clinical decision making was not always available. This could introduce delays in the pathway of care.

*You don’t have necessarily all the specialisms you need represented, so you need an extra specialism, that person may or may not be on call. There is a delay in getting that input (Patient group 1).*

A lack of continuity of clinical care over the weekend, and the likelihood that consultants would be reviewing patients who did not sit within their own primary speciality, was also problematic for clinical decision-making. For patients admitted at the weekend, their allocation to a specialist team could be delayed until the Monday. This could result in a sense of temporary responsibility in the clinicians who were caring for them over the course of one or two days at the weekend. Breaks in continuity over the weekend were also seen as impacting negatively on the management of patients who had been admitted prior to the weekend, when their care was handed over to staff who were unfamiliar with them and their history. Doctors reported that lack of knowledge of patients they saw at the weekend, and the temporary nature of their responsibility for these patients, left them reluctant to make significant decisions about implementing treatment, or changing management plans. As a result, patients were often ‘held’ on the AMU or on medical wards over the weekend without their care progressing.

*You kind of look at a set of notes and go, ‘Well, I wouldn’t have done that’, but then, ‘cause that plan’s been made by a colleague […] you’re not going to interfere with that. That is also what I find difficult about the weekend, seeing a lot of patients that you didn’t know or you’re meeting for the first time. (Clinician focus group)*

*It’s more like a babysitting service over the weekend. (Clinician focus group)*

Important decisions about patient care plans, for example, around palliative care and discharge, could be delayed due to lack of responsible senior decision-maker availability over the weekend. The speed and quality of decision-making could also be significantly compromised at the weekend by lower capacity in diagnostic and support services, including microbiology laboratory testing, scanning services and x-ray, which could introduce delays in detection of problems, diagnosis, and decision-making about treatment, and increase length of stay.

*There may be one or two slots [for scans] for the whole weekend, for the most urgent of patients, and you have to beg, barter, plead, to get your patient seen. And if […] they’re not going to get that scan until Monday, that could hinge their treatment in many directions. So the whole process is delayed by two days (Junior doctor interview).*

There were also frustrations in attempts to implement treatment decisions, and put in place therapy in order to aid patient recovery at weekends. Access to medication could be delayed due to availability of pharmacy staff, and support for therapy and for basic needs was commonly seen as more difficult to access due to fewer allied health professionals and core therapy teams at the weekend.

*We don’t have many physiotherapists, we don’t have many pharmacists, we don’t many dieticians, and there’s all those services are absolutely vital. (Clinician focus group)*

*But we can’t start an NG [nasogastric] feed without the dietician […] So we might do the swallowing assessment and say ‘yeah, you can have a soft puree’, but if they fail it, we can’t set up a feed (Clinician group 2).*

Hospital discharge was also impeded at weekends, sometimes because of lack of senior decision-making or delays from lack of in-hospital services such as pharmacy, but more usually because of non-availability of key support services in the community. Participants suggested that for some patients, particularly those who were frail or had complex needs, the community services they needed were only accessible during the week. In such cases they had to wait in an acute hospital bed until the weekend was over.

*If you want to get proper community physios [physiotherapists] you’re not going to get that over the weekend, or occupational therapy in the community, or social worker stuff. So there’s a lot of things that doesn’t happen at the weekend (Consultant interview)*

*When you have an elderly frail lady, or man, 80 plus, 90, who requires [a] package of care [in the community], […] you have to wait until Monday to get it started (Consultant interview).*

Delays in clinical decision-making, and impeded progress, were seen as problems which would not necessarily contribute to mortality. These delays did, however, have the potential to result in negative outcomes for certain patient groups, impeding patient recovery and resulting in increased length of stay. This meant increasing patients’ exposure to iatrogenic harm from prolonged hospital stays, including deterioration from immobility, deep vein thrombosis, falls, or infection.

*The longer we keep them inpatient we are risking C. diff [clostridium difficile] and hospital-acquired or healthcare-acquired infection. You can argue yes, the longer they stay, the [more] likely they will contract one of those (Clinician Group 2).*

Impediments to patient flow had consequential upstream effects. Clinicians suggested that at the weekend, more acute beds were occupied with patients whose progress along the care trajectory had slowed, resulting in ‘overflow’: patients being delayed in the Emergency Department, and new admissions being more likely to end up as outliers (patients who are admitted to a ward that does not match the specialty for treating their primary conditions). Evidence suggests [31], and focus group participants believed based on their experiences, that patients who are outliers are at higher risk of poorer quality of care and poorer outcomes.

*So if there’s a blockage somewhere, obviously that will impact on the flow as a whole, if that makes sense, so it ends up, a lot of outliers, and sometimes unnecessary prolongation of hospital stay (Consultant interview).*

### Resilience and risk of error

In our focus groups and interviews, staff and patients consistently regarded the weekend environment as being less ‘resilient’, particularly on medical wards outside the AMU. The reduced number of staff across all roles in hospitals at the weekend was seen as acting as a direct threat to safety and quality by increasing the risk of error, and reducing the extent to which procedures could be properly followed and checks performed consistently. Lower nurse staffing levels, and the increased use of bank nurses at weekends, were regarded as compromising patient safety. Concerns arose about the higher reliance on junior doctors and locums at weekends, coupled with a lack of senior executive oversight and support, which together posed a risk of poor quality decision-making about patient care, and greater potential for error.

*Obviously the less experienced team that you have, the more likely you’ll get errors. (Consultant interview).*

Overall participants felt that staff were less able to work together effectively as teams and coordinate their actions at the weekend. Handovers and communication of information were seen as weaker over the weekend due to stretched staffing and breaks in continuity; important information was not always communicated, and things could be missed.

*On the weekends when I was working you’d have a big list [handed over] and there’s all the chasing bloods. […] There was one [task on the list] I had that was literally ‘chasing FBC’ [full blood count], […] it was getting on to late in the day that I was getting round to it […] there was no information to let me know that that was a potentially urgent blood chase (Clinician focus group).*

The focus groups also expressed concerns about the lack of medicine reconciliation at weekends, due to reduced pharmacy ward cover available on medical wards, adversely affecting patient safety:

*I’ve had a Datix [incident report] with a patient that had the wrong medicines prescribed [on AMU], on a Saturday I think, got moved to a ward that didn’t have pharmacy cover and [only had] meds recs [medication reconciliation] done three days later. [They] had problems because they’d been having the wrong medications for those days and I can’t imagine that’s the only patient that’s happened to (Clinician group 2).*

Patients and relatives on wards described feeling less able to actively raise concerns or talk to a doctor about their worries during the weekend. Relatives also felt it could be more difficult for them to find out what was going on at weekends, frustrating their efforts to act as advocates for patients in ensuring that errors were not made, and that treatment was safe and appropriate.

*You can’t get the information as a relative on a ward on a weekend in the same way as you can get that information [in the week] On the basis that [the relative is] really trying to make sure somebody’s given the right treatment, it’s really infuriating not to be able to get that information. (Patient group 1).*

## Discussion

This study examined clinician and patient perceptions of the quality and safety of care for acute medical patients at weekends. Clinicians and patients alike described weaknesses in the systems and resources for delivery of care over weekend, including lower levels of staffing and differences in expertise and skill mix; breaks in continuity of care at the weekend; lack of availability of diagnostic, therapeutic and allied services; and a reduced availability of community services to support discharge. Our study makes a new contribution to the literature on weekend care in hospitals by revealing how these features of service organisation and delivery at the weekend impact on the quality and safety of care along the acute care pathway. Participants described how, in the context of stretched resources at the weekend, the primary imperative of acute care to rescue and stabilise acutely sick patients takes precedence over other functions. When fewer staff are available, the notion of effective triage and response becomes challenged, and the focus shifts to prioritisation of the sickest and most unstable patients. Both clinicians and patients felt that, while there were some challenges at the weekend in responding to the sickest patients – such as those arising from limited availability of specialist expertise and unfamiliarity of staff with the areas they were working in, on the whole patients who were very sick would receive a similar level of care at the weekend as during the week. In fact, other literature reports more positive ratings for patient experience of doctors and nurses, care and treatment, and communication, in the emergency department at weekends [26], as well as more reliable completion rates for initial vital signs measurements at weekends [8], reinforcing our findings that the quality of emergency care for the sickest patients may not necessarily be compromised at the weekend.

Participants suggested, based on their experiences, that the limited overall staffing numbers across all roles and grades at weekends meant that fewer resources were directed at formal and informal monitoring of patients who were seen as less acutely sick, or as relatively stable. This predominantly related to patients on medical wards as opposed to the acute medical unit, and included patients admitted at the weekend who had been stabilised, as well as patients who had been in hospital prior to the weekend and who had not been flagged as needing review. Participants felt there was a risk that, when patients deteriorated, this would not always be picked up and responded to quickly. Because of the lower levels of staffing and the demand on doctors at the weekend, escalation of deteriorating patients could involve more delays at the weekend. Failure to rescue has been found to be associated with reduced staffing levels and staff inexperience [32–34], and has also been identified as an explanatory mechanism for variation between hospitals in mortality rates [35].

Moving patients along a trajectory of care was seen as a particular challenge at the weekends: breaks in continuity of the team caring for the patient meant that important decisions were often delayed until after the weekend. This has resonance with previous findings that completing tasks during the ‘out of hours’ period can be challenging for staff [36], and that delays in care processes are common for acute medical patients at weekends, including those with myocardial infarction [37]. Our study suggests that delays in care are a salient feature of weekend care for both patients and staff. Along with delays in initiating treatment, making and enacting decisions about discharge were also seen as more difficult to achieve over the weekends due to lower levels of staffing, but more importantly, due to a lack of therapeutic and allied services, and an inability to arrange assessments or to set up packages of care in the community. Our study suggests that the dual goals of acute care - the ‘rescue’ and ‘disposal’ of patients - come into tension at the weekend. In the context of stretched resources, decisions about prioritisation favour rescue; this is compounded by a lack of infrastructure to support resolution of problems and patient discharge. Difficulties in moving patients on then have upstream effects on the capacity and function of the hospital. While the problem of ‘disposal’ remains pertinent across the week, it is more acute at weekends.

Our study contributes to the debate on the ‘weekend effect’ and the impact of reduced staffing levels on patient outcomes. Our findings highlight the ways in which the function and resilience of acute care can be compromised at the weekend, and risk of errors and omissions heightened at the weekend; this is reflected in evidence showing risks of hospital acquired conditions and ‘never events’ to be higher for weekend admissions [35, 36]. Our study participants argued, based on their own experiences, that detriments in quality and safety at the weekend will impact differently on different cohorts of patients and depending on patients’ stage in the care pathway, are more likely to be experienced by patients who are less acutely ill, and will impact not just on patients who are admitted at weekends, but also on those who are in hospital over the weekend.

This study is limited in that our participants were recruited from three hospital trusts in the UK, although we selected participating trusts to ensure diversity in terms of size, organisation, and characteristics of the population served. Our sample included clinicians with a range of roles including consultant, junior doctors, and nursing staff. We also included the views of PPI representatives and patients who had experienced care in hospital at weekends. Our study focused specifically on quality and safety of care in hospitals at the weekend, and we did not explore wider system issues that could impact on weekend care, for example, experiences of community services at the weekend. The data comprises participants’ accounts of their experiences, and views of weekend care, as opposed to objective measures, or observations, of weekend care. Nevertheless we suggest it provides important insights into aspects of care delivery, quality, and safety for acute medical patients who are in hospital at weekends. The wider HiSLAC [High intensity specialist-led care] study includes analysis of outcomes data, case note review, and extensive ethnographic observations, which will allow us to verify and extend these findings, and test hypotheses about quality and safety of care at weekends.

## Conclusions

Focusing on explaining the reasons for increased mortality for weekend admissions risks obscuring important questions about the quality and safety of care for all patients in hospital at weekends. While detriments in staffing and resources may not ultimately result in excess mortality, the consequences in terms of quality and safety, and patient experience of care, are nonetheless undesirable. These consequences will impact both on patients admitted at weekends, and those whose stay includes a weekend. Wholesale restructuring of hospitals, and increased weekend staffing, to provide full seven day services may not be feasible, effective, or cost effective. Our findings suggest the value of focusing instead on care processes and systems resilience over the weekends, and how these can be better supported, even in the limited resource environment that exists in many hospitals at weekends. This may require considering: how work is prioritised at the weekend; how continuity and momentum can be maintained at the weekend when teams are broken up and the patterns of staffing, and mix of expertise changes; how robust systems for detecting and responding to deteriorating patients can be maintained at weekends; how hospital diagnostic and therapeutic services can more effectively support active patient management; and the extent to which social and community services are configured to help avoid weekend hospital admissions for vulnerable patients, and to enable timely and safe discharge. Learning from examples where hospitals have been able to protect quality and safety of care at weekends, for example, using a positive deviance approach [38], will help inform the development of feasible and effective approaches to improving weekend care.

## Additional files

Additional file 1: HiSLAC v2 Focus group- topic guide - clinicians. Topic guide used in clinician interviews and focus groups. (DOCX 25 kb)

Additional file 2: HiSLAC focus group - topic guide -patients. Topic guide used in patient interviews and focus groups. (DOC 61 kb)

Additional file 3: The weekend effect FGs round 2 clinicians 251016. Supplementary material for clinician interviews and focus groups. (PDF 485 kb)

Additional file 4: The weekend effect FGs round 2 patients 181016. Supplementary material for clinician interviews and focus groups. (PDF 401 kb)

Additional file 5: Descriptive coding frame used for initial coding of data. (DOCX 40 kb)

## Abbreviations

A&E: Accident & emergency
AMU: Acute medical unit
C. diff: Clostridium difficile
FBC: Full blood count
HiSLAC: High intensity specialist-led care
Meds recs: Medication reconciliation
NG: Nasogastric
Physio: Physiotherapist
PPI: Patient and Public Involvement
Resus: Resuscitation area
