# Supplementary material for: Quality and safety of in-hospital care for acute medical patients at weekends: a qualitative study
Source: BMC Health Serv Res. 2018 Dec 29;18:1015. doi: 10.1186/s12913-018-3833-z (PMC6310936; doi:10.1186/s12913-018-3833-z)
Supplement: Supplementary file 1 — HiSLAC v2 Focus group- topic guide - clinicians. Topic guide used in clinician interviews and focus groups. (DOCX 25 kb) [file 12913_2018_3833_MOESM1_ESM.docx]

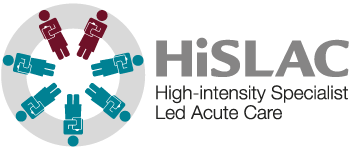


| **Group** | **Clinicians** |
| --- | --- |
| **Date of focus group** |  |

**Preamble**

- *The facilitator will introduce themselves, including their position within the HiSLAC project.*
- *Explain the objectives of HiSLAC and the focus group.* Our plan today is to provide you with some information about what the literature from our study has found so far before asking you what you think and how well you feel your experiences are the same or different to those outlined
- *Ensure that participants have read and understand the Information Sheet and have been given the opportunity to ask any questions.*
- *State we will digitally record the focus groups so that we capture the thoughts, opinions, and ideas from the group. No names will be attached to the focus groups and the recordings will be destroyed as per University of Birmingham guidelines.*
- *Remind participants that all information will be treated in confidence and taking part in the focus group is voluntary.*
- *Remind participants to please keep any information shared in the group confidential, and not discuss outside the focus group.*
- *Time Frame - Inform participants that the focus group is likely to take around 2 hours and lunch will be provided after the meeting.*
- *Iterate that the HiSLAC team will either email or post a one page summary with the key themes/topics and search terms generated from the focus group. Participants will then be asked to comment if any important areas have been excluded from the summary.*
- *Inform participants that the role of the facilitator is to guide the discussion and that there are no right or wrong answers, only differing points of view.*

Thank you all for coming today. We really appreciate you taking the time to do so.

**Warm up questions**

**Clinicians**

1. Can you tell me how long have you been working in the NHS and your job title?
2. What department do you work in?

Show slides all the way through

**Ok – now I’m just going to take you back through the slides and ask you about each factor that we have identified**

**We would like to hear about your experiences and opinions, and the extent to which you feel each factor is a problem in reality and how this impacts on patients.**

**You might not feel that some of these issues are a problem at weekends, based on your own experience, and if so we’d be interested to hear that too.**

**For each slide, ask:**

How well does this description fit with your experiences?

In your experience, does this factor impact on patient care and patient outcomes? Can you explain how and why?

Can you give any examples from your own experience?

Is there anything about this factor you disagree with, or you feel needs to be qualified or expanded?

Is there anything else important related to this factor that we haven’t identified? (e.g. for staffing levels – is this about number of consultants per se, or does the type of consultant matter? What about junior doctors? What about other staff e.g. admin?)

How important do you think this factor is in explaining the weekend effect? (ask them to rate each factor out of 10?)

How easy is it to improve this factor at weekends? What needs to be considered in any efforts to improve things in relation to this factor?

**At the end**

Is there anything else you think is important?

Is there anything you think mitigates against the weekend effect, or helps make care better at the weekend?
