# Supplementary material for: Quality and safety of in-hospital care for acute medical patients at weekends: a qualitative study
Source: BMC Health Serv Res. 2018 Dec 29;18:1015. doi: 10.1186/s12913-018-3833-z (PMC6310936; doi:10.1186/s12913-018-3833-z)
Supplement: Supplementary file 2 — HiSLAC focus group - topic guide -patients. Topic guide used in patient interviews and focus groups. (DOC 61 kb) [file 12913_2018_3833_MOESM2_ESM.doc]

| 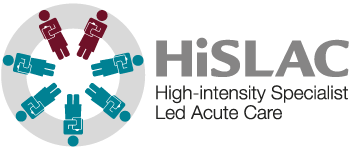 | |
| --- | --- |
| **Focus Group - Topic Guide** | |
| **Group** | **Patients** |
| **Date of focus group** |  |

**Preamble**

- *The facilitator will introduce themselves, including their position within the HiSLAC project.*
- *Explain the objectives of HiSLAC and the focus group.*
- *Ensure that participants have read and understand the Information Sheet and have been given the opportunity to ask any questions.*
- *State we will digitally record the focus groups so that we capture the thoughts, opinions, and ideas from the group. No names will be attached to the focus groups and the recordings will be destroyed as per University of Birmingham guidelines.*
- *Remind participants that all information will be treated in confidence and taking part in the focus group is voluntary.*
- *Remind participants to please keep any information shared in the group confidential, and not discuss outside the focus group.*
- *Time Frame - Inform participants that the focus group is likely to take around 2 hours and lunch will be provided after the meeting.*
- *Iterate that the HiSLAC team will either email or post a one page summary with the key themes/topics and search terms generated from the focus group. Participants will then be asked to comment if any important areas have been excluded from the summary.*
- *Inform participants that the role of the facilitator is to guide the discussion and that there are no right or wrong answers, only differing points of view.*

Thank you all for coming today. We really appreciate you taking the time to do so.

**Introductions/warm up questions**

1. If we could go round and introduce ourselves by first name?
2. How did you come to take part in this focus group?

Show slides

**Stop at last slide**

**These are a set of ideas from clinicians, patients and researchers about the difference between weekends and weekdays, and these may or may not match your own experiences.**

**Talk through the last slide**

**Ok – now I’m just going to ask you for your views and experiences about whether you have experienced differences between the weekend and the weekday in hospital, and whether this affected your treatment or care in any way.**

**You might not feel that some of these issues are a problem at weekends, based on your own experience, and if so we’d be interested to hear that too.**

**Your experience is what we’re interested in.**

Have you noticed any differences between care at the weekend and the week in relation to any of these issues that we have identified?

Clarify which issue, ask about their experiences.

Has this particular issue made any difference to your care and treatment?

Explore in what ways - Can you give any examples?

What impact did that have?

If they disagree with any factors, ask them to describe their experiences / explain.

Did you experience any delays in your care? Clarify if in week or weekend

Explore where this was and why it happened?

What impact do you think that had?

**Overal**l

Is there anything else that you have found to be different at the weekend compared to the week?

To what extent did this matter to you?

What difference did it make to your care and treatment?

What would you improve and how?

**Concluding remarks**

- Thank participants again for their participation in the focus group and provide voucher
- Reiterate that participants will only be acknowledged by name if they consent to do this and give sheet for signature
