# Supplementary material for: Quality and safety of in-hospital care for acute medical patients at weekends: a qualitative study
Source: BMC Health Serv Res. 2018 Dec 29;18:1015. doi: 10.1186/s12913-018-3833-z (PMC6310936; doi:10.1186/s12913-018-3833-z)
Supplement: Supplementary file 3 — The weekend effect FGs round 2 clinicians 251016. Supplementary material for clinician interviews and focus groups. (PDF 485 kb) [file 12913_2018_3833_MOESM3_ESM.pdf]

HiSLAC
High-intensity Specialist Led Acute Care

University of Leicester

The weekend effect:

What we know and don't know from the literature

The weekend effect and the HiSLAC project

- More people die following admission to hospital at the weekend compared to when they are admitted during a weekday. Are patients sicker at weekends or is something else going on?
- The HiSLAC project is looking at the way that care for acute medical patients is organised at weekends in 20 different hospitals in England to find out why care might differ at weekends compared to in the week, and whether this might result in increased risk of mortality.
- We are conducting observations and interviews with staff in the hospitals, and undertaking a literature review.

Possible contributing factors to the weekend effect

- Based on initial focus groups with clinicians and patients, and evidence from the literature we have identified some possible factors that could explain the weekend effect

- We would like to explore your views of each of these factors in terms of how and why it could contribute to the weekend effect, and your opinions on how important each factor is in explaining the weekend effect

HiSLAC High-intensity Specialist Led Acute Care

University of Leicester

Staffing levels

- Fewer consultants available at weekends
  - -> less proactive monitoring and review of patients, risk of deterioration
  - -> delayed / poorer quality decision-making (diagnosis and treatment)
  - -> delays in escalating patients / decisions about end of life care
  - -> less supervision of junior staff
  - -> delayed discharges
  - -> ?more medical errors

- Fewer nurses / less well qualified nurses
  - -> poorer quality of care

Handover / communication poorer at weekends

- Lack of standardised handover
- Evidence of a lack of standardisation of handover notes and located in different places at weekends
- Less continuity of staff
- More clinical uncertainty – doctors lack enough of the right information to make decisions
- Care plans not effectively discussed between different levels of staff at weekends

Lack of availability of in-hospital allied services

- Diagnostics / therapy / nutrition / pharmacy
    - -> delays to diagnosis and treatment
    - -> patient deterioration
    - -> delayed discharge
    - -> ?medication errors

Patient flow is impeded at weekends

- -> delayed discharges (decision-making, availability of in-hospital and community services)
- -> changes in pattern of demand at the weekend
- -> ?outliers and transfers more of a problem

Patients admitted at weekends are different

- Patient demand is different at the weekend
- Patients are sicker (and therefore more likely to die). Some evidence for this:
  - Excess mortality patterns of the weekend effect vary widely for different diagnostic groups (Concha et al 2013) .
  - Evidence that patients are sicker (NEWS scores) than on weekdays, although scores were recorded earlier compared to those admitted during the week. After adjusting for levels of sickness, patients were at no greater risk of death than those admitted during the week (Mohammed 2016).
