# Supplementary material for: Quality and safety of in-hospital care for acute medical patients at weekends: a qualitative study
Source: BMC Health Serv Res. 2018 Dec 29;18:1015. doi: 10.1186/s12913-018-3833-z (PMC6310936; doi:10.1186/s12913-018-3833-z)
Supplement: Supplementary file 4 — The weekend effect FGs round 2 patients 181016. Supplementary material for clinician interviews and focus groups. (PDF 401 kb) [file 12913_2018_3833_MOESM4_ESM.pdf]

HiSLAC
High-intensity Specialist Led Acute Care

University of Leicester

The weekend effect:

What we know so far…

The weekend effect and the HiSLAC project

- More people die following admission to hospital at the weekend compared to when they are admitted during a weekday
- The HiSLAC project is looking at the way that care for acute medical patients is organised at weekends in 20 different hospitals in England to find out why care might differ at weekends compared to in the week, and how this might result in increased risk of death.
- We are conducting observations and interviews with staff in the hospitals, and undertaking a literature review.

The aims of this discussion

- Based on initial focus groups with clinicians and patients, and evidence from the literature we have identified some issues that could help to explain the weekend effect

- We would like to explore your views about how and why these issues may contribute to the weekend effect, and any experiences of your care that you may want to share

HiSLAC
High-intensity Specialist Led Acute Care

University of Leicester

Issues that may contribute to the 'weekend effect'

- There are fewer consultants and nurses available at weekends
  - Affects decision-making, treatment planning and quality of care
- Communication is worse at weekends
  - patient/carer information not forthcoming, doctors lack the right information, different staff on duty
- Lack of availability of in-hospital services
  - Diagnostic tests, therapy, nutrition, pharmacy
- Problems with discharge, and fewer beds available; patients can end up on the wrong wards
- More and different types of patients admitted at the weekend - sicker
