# Supplementary figures and images for: Quality and safety of in-hospital care for acute medical patients at weekends: a qualitative study
Source: BMC Health Serv Res. 2018 Dec 29;18:1015. doi: 10.1186/s12913-018-3833-z (PMC6310936; doi:10.1186/s12913-018-3833-z)

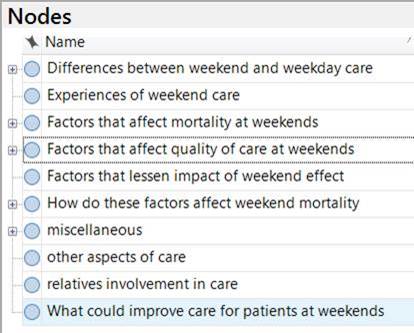

Supplement: Supplementary file 5 — Descriptive coding frame used for initial coding of data. (DOCX 40 kb) [file 12913_2018_3833_MOESM5_ESM.docx]
